# Elective single embryo transfer- the power of one

**DOI:** 10.1186/s40834-016-0023-4

**Published:** 2016-07-06

**Authors:** Amy M. Lee, Matthew T. Connell, John M. Csokmay, Aaron K. Styer

**Affiliations:** 1 Massachusetts General Hospital Fertility Center, Vincent Memorial Obstetrics and Gynecology Service and Harvard Medical School, Boston, MA 02114 USA; 2 Department of Obstetrics, Gynecology, and Reproductive Biology, Harvard Medical School, Boston, MA 02114 USA; 3 Walter Reed Army Medical Center, Washington, DC USA; 4 Program in Reproductive and Adult Endocrinology, NICHD, National Institutes of Health, Bethesda, MD USA; 5 Vincent Reproductive Medicine and IVF, Vincent Department of Obstetrics and Gynecology, Massachusetts General Hospital, Yaw 10A, 55 Fruit Street, Boston, MA 02114 USA

**Keywords:** Infertility, In-vitro fertilization, Elective single embryo transfer, Twin gestation assisted reproductive technology

## Abstract

Despite the highest historical live birth success rates for couples undergoing in vitro fertilization (IVF), there has been an epidemic of iatrogenic twin and higher order gestation conceived from this treatment. Continued improvement in cryopreservation techniques have allowed preservation of supernumerary embryos for use in future cycles, and refinements in culture systems and embryo selection have resulted in the transfer of fewer embryos while maintaining favorable pregnancy rates. The voluntary transfer of a single high quality embryo, elective single embryo transfer (eSET), has significantly reduced multiple gestation rates and maximized the rate of singleton pregnancy without compromising overall success rates. Although eSET is the standard of care in several developed countries, utilization in the United States has been slow. States with mandated IVF insurance have seen decreases in preterm birth rates yielding down stream health care savings. Herein, the evolution and future applications of this practice to reduce the risk of iatrogenic twins is reviewed.

## Background

Since its inception in 1978, in vitro fertilization (IVF) has resulted in nearly 4 million births. Initially, IVF utilized unstimulated, natural cycles with laparoscopic oocyte retrievals and was fraught with inefficiencies and low pregnancy rates. The subsequent use of ovarian stimulation with exogenous gonadotropins allowed for the selection of several dominant ovarian follicles, retrieval of multiple oocytes, and improved pregnancy rates. In the mid-1980s, published pregnancy rates documented a success rate of 20 % with one embryo, and up to 40 % pregnancy rate per IVF attempt with the transfer of four embryos [1]. Multiple embryos were transferred to maximize pregnancy rates. As a result, the debate surrounding the ideal number of embryos for transfer was born and has been ongoing for the past three decades [2].

In the early 1980s, the rise in Advanced Reproductive Technologies (ART) success rates was synonymous with the transfer of multiple embryos and multiple gestation pregnancy. The first report of IVF twins in 1981 was rapidly followed by abundant reports; multiple gestations and IVF became forever conjoined [3, 4]. Multiple gestations are wrought with substantial perinatal and neonatal complications: pregnancy loss, preterm births, congenital abnormalities, and increased perinatal mortality [5]. Likewise, the financial impact from the health care costs of multiple gestations has persisted through the decades. In the early 1990s when the incidence of multiple gestations from IVF was 15–30 %, the delivery-related hospital expenses was up to four times more per child for multiple gestations compared to singletons [6]. From 1998 to 2011, there has been a 29 % decrease in the number of multiple gestations and 33 % decrease in higher order multiple gestations. However, the rate of twin gestation has plateaued and 36 % of all twins and 77 % of higher order multiples are still due to ART [7]. As a result, annual expenditures for iatrogenic preterm deliveries totals twenty-six billion dollars of healthcare costs [8, 9].

## Methods

A comprehensive online search of peer reviewed published literature in Entrez Pubmed (US National Library of Medicine, National Institute of Health; http://www.ncbi.nlm.nih.gov/pubmed/) was conducted for content related to the utilization of single embryo transfer in IVF therapy. References for this review were identified with the use of the following terms: infertility, in-vitro fertilization, elective single embryo transfer, and twin gestation assisted reproductive technology. Additional references were also identified in the bibliographies of articles identified in the primary query. The reference lists of included articles were also reviewed to identify additional relevant studies. Studies were included if they met the following criteria: the study population included women of reproductive age undergoing IVF or intracytoplasmic sperm injection (ICSI) in nondonor cycles; [2] the number of embryos transfers was recorded for all participants; [3] the clinical outcome of implantation and/or clinical pregnancy was recorded; and [4] any study design except case reports.

## Introduction of elective single embryo transfer

In the late 1990s, significant improvements in IVF pregnancy success rates challenged the mantra that the transfer of higher numbers of embryos were required to obtain acceptable pregnancy rates. In 1998, Templeton et al. demonstrated that transferring four embryos resulted in significant increases in multiple gestations without improvements in pregnancy rates when compared to three embryos [10]. In 1999, Gerris et al. were the first to evaluate elective single embryo transfer (eSET). Elective single embryo transfer (eSET) is the intentional transfer of one embryo when there are multiple embryos of appropriate stage and quality available. This should be differentiated from obligatory or non-elective single embryo transfer, where the patient has only one embryo available for transfer. This group randomized women less than 34 years of age to either eSET or double embryo transfer (DET). Although the implantation rate was the same between the groups (relative risk [RR] 0.88, 95 % CI 0.52–1.49), the ongoing pregnancy rate was 35 % higher than the DET group. Overall, the multiple gestation rate in the eSET group was dramatically reduced compared to the DET and were comparable to naturally-conceived pregnancies [11]. Subsequent studies demonstrated similar clinical pregnancy rates between elective single and double embryo transfer [12, 13].

In an effort to minimize the medical and financial risks of multiple gestations, the Belgium government passed legislation that allowed for federal reimbursement of embryology laboratory expenses conditional on low number embryo transfer. Effective July 1, 2003, women up to age 36 were only allowed a single embryo transfer. In women between ages 36 and 39, a double embryo transfer could be performed; no limitations were placed on women older than 39 years of age. In a retrospective review of the first six months after the enactment of this legislation, the utilization of eSET markedly improved from 14 to 49 %, overall pregnancy rates remained stable, and the twin pregnancy rate declined significantly [14].

## Cryopreservation and elective single embryo transfer

Development and improvements of embryo cryopreservation techniques has allowed supernumerary embryos to be retained and utilized in subsequent cycles if the first cycle is unsuccessful [15, 16]. Thurin et al. assessed the cumulative effect of fresh and subsequent thawed embryo transfer on eSET success rates in a multi-centered randomized trial. This study included 331 women less than 36 years of age, with two good-quality embryos during their first IVF attempt. Subjects were randomized to either single-embryo transfer followed by single frozen-and-thawed embryo transfer (assuming no live birth) or to double embryo transfer. They found no statistically significant difference in live birth rate when comparing cumulative single embryo versus double embryo transfer rate (38.8 % vs. 42.9 %, *P* = 0.3). Compared to the eSET arm, the rate of multiple births was significantly greater in the double embryo group (0.8 % vs. 33.1 %, *P* < 0.001). Over the last decade both observational and randomized studies have consistently observed similar rates of live birth rates in eSET compared to DET, with a significant reduction in twin pregnancy rates with eSET [17, 18].

## Embryo selection

With extension of the duration of embryo culture from 2 to 3 days to 5 days (blastocyst stage) embryo(s) with the highest implantation potential may be selected for transfer. Improvements in implantation rates were observed and single blastocyst transfers were found to have superior pregnancy rates compared to single or double cleavage stage (day 2 or 3) transfer [19, 20]. Single day 5 blastocyst transfer was found to have similar live birth rates as those with two blastocysts transferred, and only half the multiple gestation rate [21, 22]. High quality blastocysts (defined by the 2010 Society for Assisted Reproductive Technology (SART) grading system based on morphology, expansion and overall quality) resulted in significantly higher rates of live birth compared to use of lesser quality embryos [23].

## Preimplantation genetic screening’s role (PGS) in eSET

The transfer of a single embryo requires choosing the embryo with the best chance of implanting and progressing to a live birth. Even transfer of a known euploid embryo does not guarantee a live birth. Traditionally, embryologists select an embryo for transfer based on morphologic characteristics though this is not without error. It has been shown that approximately 20 % of day 5 embryos transferred based on this method alone may be aneuploid [24].

The transfer of a single biopsy tested euploid embryo may overcome the age related decline in success. The use of PGS provides an important tool in our armamentarium for embryo selection. This may benefit patients of advanced reproductive age by allowing for greater success of a single embryo transfer; particularly as multiple gestation pregnancies may carry higher risks for these patients. Forman et al. randomized 175 patients aged 43 and less to the transfer of 1 euploid embryo versus two unscreened embryos and found that pregnancy rates were no different (69 % after euploid eSET and 72 % after untested DET, *P* = 0.6). Furthermore, this SET resulted in fewer twin pregnancies, preterm deliveries, and NICU admissions [25]. These pregnancy and neonatal outcomes are now considered the standard for IVF success and reflect the emphasis shift to provide safer and healthier outcomes for our patients.

## Candidates for eSET

Initially elective single embryo candidates were defined as female patients less than 34–36 years old with numerous ‘good quality’ embryos. The most current eSET evidence demonstrates marked reduction in multiple gestations and better neonatal outcomes without compromising live birth rates. While the benefits are clearly present, there exists the challenge of implementing an effective eSET protocol and choosing appropriate patients for this policy. Female age is one of the strongest predictors of success in IVF and should be weighed in any future eSET policies [26].

However, there exists some controversy as to which age eSET should be offered or recommended. The American Society of Reproductive Medicine practice guideline from 2013 stated that good prognosis patients less than 35 years old have a single day 5 embryo transferred (Table 1). While patients above the age of 35 reasonably may be offered transfer of 2 or 3 embryos [27]. Since this publication, there has been considerable work done on patients older than 35 with regards to eSET that suggest that these guidelines may need revision. In examining patients 38 and younger, van Montfoort et al. compared those with good quality cleavage stage embryos who underwent eSET versus those who received DET. There were no significant differences in the first, second, or third cycles in regards to ongoing pregnancy rates. Furthermore, patients receiving eSET had more embryos to freeze and thus had higher cumulative pregnancy rates [28]. Veleva et al. followed up with a study in patients aged 36–39 years of age. Patients with top quality embryos who underwent eSET had similar pregnancy rates per embryo and similar live birth rates when compared to DET. Again it was demonstrated that patients who had embryos frozen had higher cumulative pregnancy rates (54.0 % versus 35.0 %, *P* < 0.0001) live births (41.8 % vs 26.7 %, *P* < 0.0001) [29]. These data should be interpreted with caution, as patients receiving eSET in both studies were only good prognosis patients.

**Table 1:** Recommended limits on the numbers of embryos to transfer

Prognosis	<35	Age (yr) 35–37	38–40	41–42
Clevage- stage embryos				
Favorable^a^	1–2	2	3	5
All others	2	3	4	5
Blastocyts				
Favorable^a^	1	2	2	3
All others	2	2	3	3

^a^Favorable = first cycle of IVF, good embryo quality, excess embryos available for cryopreservation, or previous successful IVF cycle

Reprinted from *Fertil Steril*, 99/1, American Society of Reproductive Medicine. Practice Committe Opinion. Criteria for number of embryos to transfer: a committee opinion, 44–6, Copyright (13), with permission from Elsevier [27]

While there is clear data on the success of select good prognosis patients receiving eSET below age 35, there is relatively little data on an upper age limit. Niinimaki et al. sought to determine if older women aged 40–44 years old could have reasonable cumulative pregnancy rates if an eSET policy was applied. In this retrospective study, good prognosis patients received either an eSET or DET in the fresh cycle. They were allowed a DET if subsequent frozen cycle was performed. In the fresh cycle there were no differences in clinical pregnancy or live birth rates between the eSET and DET groups. Interestingly, cumulative clinical pregnancy and live birth was higher in the eSET group, likely a reflection of the eSET group having more and better quality embryos [30].

Collectively, these data suggest the upper limit of eSET may be extended beyond the prior age guideline of 35 years. This has already been reflected in U.S. clinical practice as rates of eSET in patients aged 35–37 has increased 0.7 % per year since 2004 [31]. However, an age based eSET policy must not discount the importance of assessing patients who are of a good prognosis. van Montfoort et al. conducted a randomized controlled trial of 335 patients (<38 years old) allocated to either eSET cycle irrespective of embryo quality or eSET only for good embryo quality otherwise DET. Although cumulative live birth rates were similar (62.4 % vs 62.6 %, *P* > 0.05) they also demonstrated lower live birth rates (21.3 % vs 13 %, *P* < 0.05) in the first fresh cycle in the group randomized to eSET [32]. When reviewing SART data, patients with the highest likelihood of a successful IVF outcome in eSET regardless of age were patients receiving a day 5 embryo transfer and having 3 or more embryos to transfer, surrogate markers for improved prognosis [31]. These data support eSET in good prognosis patients less than 38 years. There likely exists a benefit of eSET in select patients older than 38, but the paucity of data requires further study to determine an upper age limit.

## Perinatal outcomes

Since IVF pregnancy rates continue to improve to their highest historical levels, there has been a significant emphasis on infant/perinatal outcomes and ART safety. Prior to the initiation of eSET, 30 % of IVF cycles resulted in multiple gestations compared to the population rate of 1.5 %. Severe maternal complications include increased rates of anemia, hypertensive disorders and pre-eclampsia, gestational diabetes, postpartum hemorrhage, and operative deliveries. The European Society of Human Reproduction and Embryology Capri Workshop in 2000 addressed the 4 to 10-fold increase in perinatal morbidity and mortality associated with twins. Multiple gestations are at increased risk of preterm delivery (particularly early preterm <32 weeks or peri-viable delivery), fetal growth restriction and intrauterine fetal demise of one or both twins. Long term morbidity also includes complications from hypoxic ischemic encephalopathy and cognitive delays [33].

Emerging evidence has demonstrated the long-term neonatal benefits for infants conceived with eSET. In a meta-analysis of studies between 1999 and 2010 which reviewed perinatal and neonatal outcomes in eSET and DET, the risk of preterm birth and low birth weight was minimized with eSET. However, no significant difference were appreciated in the rates of spontaneous abortion, early preterm birth, or perinatal mortality [34]. In a review of national data submitted to the Centers for Disease Control (CDC) from 1999 to 2010, patients who underwent single embryo transfer were twice as likely to have a good perinatal outcome when compared to those with more embryos transferred. The strongest predictor for a good perinatal outcome was utilization of elective single blastocyst transfer [31]. Analysis of the 2011 CDC data showed women with favorable prognoses (defined as having at least one embryo more available for cryopreservation) who underwent eSET, had a significant increase in term healthy neonates and 77 % reduction in preterm births [9]. These findings suggest that the benefit of single embryo transfer extends beyond preventing multiple pregnancies. Moreover, recent studies have redefined the most relevant definition of IVF success as term gestation singleton live birth [35].

## Current status

**Fig. 1:**
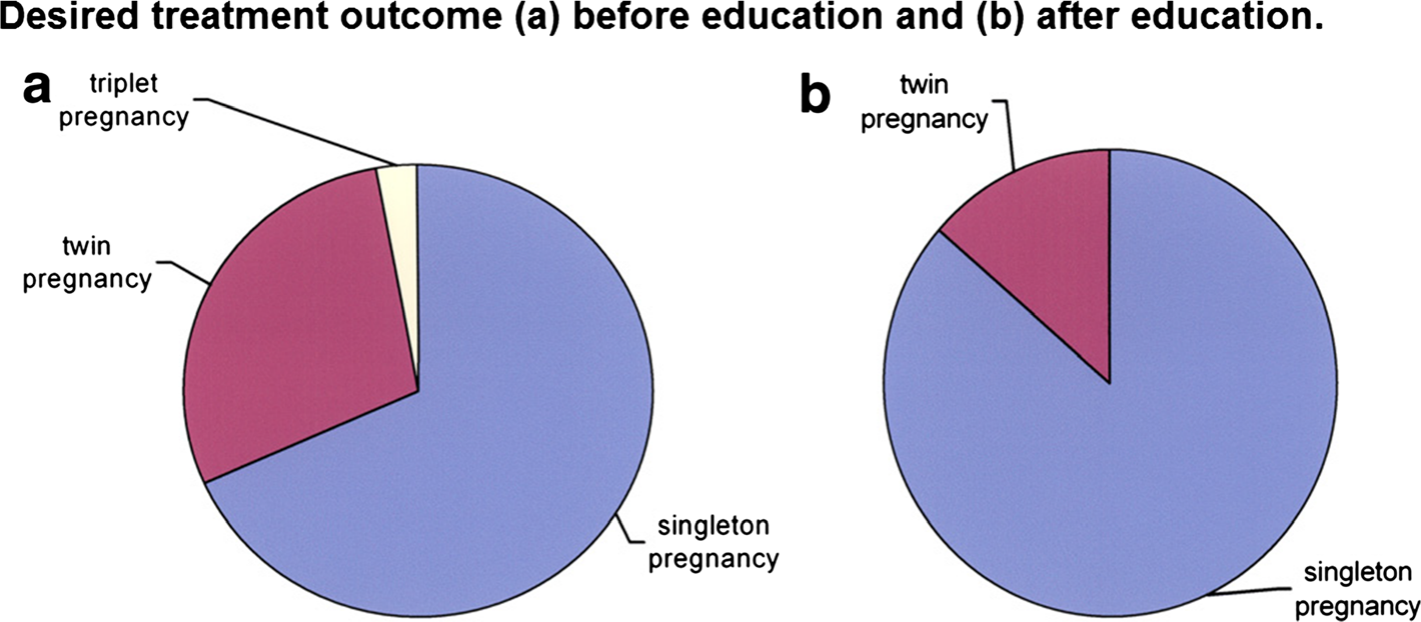
Desired treatment outcome **a** before education and **b** after education. Reprinted from *Fertil Steril*, 88/2, Ryan et al. A mandatory single blastocyst transfer policy with educational campaign in a United States IVF program reduces multiple gestation rates without sacrificing pregnancy rates, 354–60, Copyright (2007), with permission from Elsevier [39]

Compared to their non-infertile cohorts, patients with infertility are at least twice as likely to desire multiple pregnancy when compared to those without trouble conceiving [36]. It has been shown that although initially 54 % of IVF patients would prefer single embryo transfer, that rate dropped dramatically to 15 % if choosing such would reduce the chance of pregnancy by as little as 5 % [37]. Patients planning to undergo IVF treatment would prefer to undergo double embryo transfer resulting in a child with significant impairment than no child at all [38]. Ryan et al. conducted a novel study investigating the impact of their educational campaign, in the setting of an institutional mandatory single embryo transfer policy. Following education, more patients were more likely to choose single embryo transfer over double embryo transfer (if given the theoretical option). Patients preferred a singleton pregnancy as the desired treatment outcome, Fig. 1 [39].

**Fig. 2:**
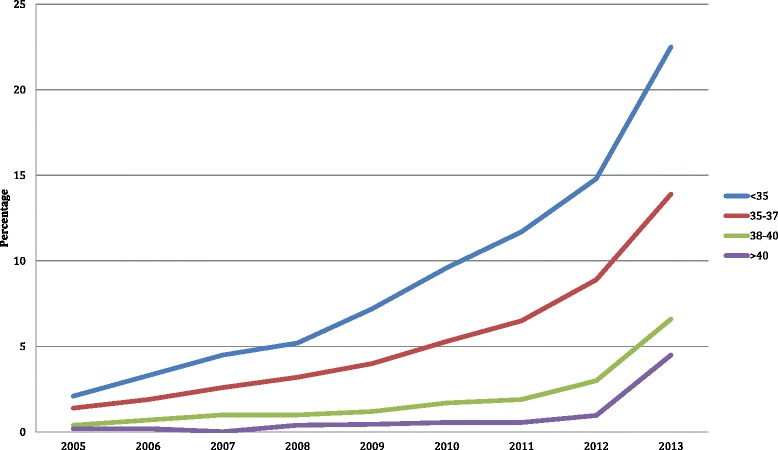
Cycles with single embryo transfer. Trends in the percentage of cycles using elective single embryo transfer, United States from 2005 to 2013. The data source for this figure was the Society for Assisted Reproductive Technology (SART) Clinic Outcomes Reporting System (CORS) database (http://www.sart.org)

According to available data, 1.5 % of lives births in the United States result from IVF and the mean cycle success of rate is 46 %. In 1992, Congress enacted The Fertility Clinic Success Rate and Certification Act that mandated the standardized reporting of success rates by all clinics providing ART. In the initial years that the data were available, 58 % of transfers in women under age 35 involved 4 or more embryos. The Society for Assisted Reproductive Technology released their first guidelines in 1998 outlining the maximum recommended number of embryos transferred. During this time, women who were less then 35 years old were limited to 3 cleavage-staged embryos while women over forty were capped at five [40]. Following serial updates of these guidelines, there was a decline in both the number of embryos transferred and higher order multiples, though the number of twin births remained static [41]. In the U.S., utilization has increased eight-fold since 2004, across all age groups [31]. Notably, the most significant rise has been in the last 3 years (Fig. 2). When compared to other developed countries, disparities persist in the utilization of elective single embryo transfer in the United States. In a review of global ART utilization, the use of eSET in the U.S. is the lowest among the countries reviewed. Consequently, ART twin birth rates are 9 % higher in the U.S. compared to Europe [42]. It is hopeful, that results such as these will serve to increase the adoption of eSET as mainstream practice when clinically indicated in the U.S.

Financial responsibility for the individual and society plays a major role in the decision of the number of embryo(s) to transfer. Cycles financed by the couple were less likely to utilize single embryo transfer than those covered by insurance [43]. Initially, studies evaluating cost effectiveness favored double over single embryo transfer, but this was likely due to the decreased efficacy of eSET at the time [44]. The adoption of fresh eSET with sequential cryothaw (if indicated) has been shown to be a cost effective approach without impacting pregnancy rates [45]. A recent retrospective cohort study of 13,000 ART-related pregnancies investigated the total expenses for the mother (including antenatal admission and delivery) and infant(s) through the first year of life during a 5-year period. The reduction of iatrogenic multiple gestations to singletons was estimated to result in a cost savings of six billion dollars annually [8].

## Mandated insurance coverage for ART

Infertile couples often have been through an arduous journey and may favor transfer of multiple embryos if there is a small increase in pregnancy rate; regardless of the risks. These motivations may include a desire to become parents as quickly as possible but also may be financially driven. To alleviate this financial burden and improved safety, several states have passed laws to make IVF a covered benefit. States that have IVF insurance coverage laws show that ART services are more frequently utilized suggesting better access to care. In addition, these same states have fewer embryos transferred per cycle suggesting that couples are more amenable to eSET when there is less financial strain [46]. States with mandated coverage have fewer high-order multiple births, fewer preterm births, and fewer low birth weight infants when compared to states without mandated coverage [47]. One province in Canada (Quebec) switched to public funded IVF clinics and their results are similar to mandated insurance states. Namely, there was greater ART utilization, increased use of eSET, and fewer twin pregnancies. Analysis showed that there was a modest increase in price of an IVF cycle but this was offset by the downstream savings from neonatal health care at 1 year [48]. These data point towards better neonatal outcomes when the financial burden of IVF is either completely or partially alleviated. Optimistically, these data will pave the way for expanding insurance coverage of IVF and support the benefit of eSET.

## Conclusion

The past three decades have seen the emergence of IVF as the gold standard treatment for infertility. The era of low live birth rates and the routine practice of multiple embryo transfer are a thing of the past. Advancement in embryo cryopreservation, extended embryo culture with blastocyst selection, and preimplantation genetic screening has facilitated the expansion of elective single embryo without compromising outcomes. Mandated infertility coverage in Europe, Canada and selected states have resulted in increased eSET utilization and decreased costs associated with ART. Moving forward, reproductive medicine should aim for the gold standard IVF outcome to be a singleton term live birth pregnancy with eSET. When the goal is to minimize IVF complications, multiple embryo transfer does not necessarily translate to a superior outcome. The future success of ART lies in elective single transfer, the power of one.

## References

[1] Muasher S, Wilkes C, Garcia J, Rosenwaks Z, Jones H (1984). Benefits and risks of multiple transfer with in vitro fertilisation. Lancet.

[2] Craft I, Porter R, Green S, Tucker M, Smith B, Twigg H (1984). Success of fertility, embryo number, and in-vitro fertilisation. Lancet.

[3] Feichtinger W, Szalay S, Kemeter P, Beck A, Janisch H (1982). Twin pregnancy after laparoscopic oocyte recovery, in-vitro fertilization and embryotransfer (author's transl). Geburtshilfe Frauenheilkd.

[4] Kerin J, Quinn P, Kirby C, Seamark R, Warnes G, Jeffrey R (1983). Incidence of multiple pregnancy after in-vitro fertilisation and embryo transfer. Lancet.

[5] Tummers P, De Sutter P, Dhont M (2003). Risk of spontaneous abortion in singleton and twin pregnancies after IVF/ICSI. Hum Reprod.

[6] Callahan TL, Hall JE, Ettner SL, Christiansen CL, Greene MF, Crowley WF (1994). The economic impact of multiple-gestation pregnancies and the contribution of assisted-reproduction techniques to their incidence. N Engl J Med.

[7] Kulkarni AD, Jamieson DJ, Jones HW, Kissin DM, Gallo MF, Macaluso M (2013). Fertility treatments and multiple births in the United States. N Engl J Med.

[8] Allen BD, Adashi EY, Jones HW (2014). On the cost and prevention of iatrogenic multiple pregnancies. Reprod Biomed Online.

[9] Kissin DM, Kulkarni AD, Kushnir VA, Jamieson DJ, National ARTSSG (2014). Number of embryos transferred after in vitro fertilization and good perinatal outcome. Obstet Gynecol.

[10] Templeton A, Morris JK (1998). Reducing the risk of multiple births by transfer of two embryos after in vitro fertilization. N Engl J Med.

[11] Gerris J, De Neubourg D, Mangelschots K, Van Royen E, Van de Meerssche M, Valkenburg M (1999). Prevention of twin pregnancy after in-vitro fertilization or intracytoplasmic sperm injection based on strict embryo criteria: a prospective randomized clinical trial. Hum Reprod.

[12] Martikainen H, Tiitinen A, Tomás C, Tapanainen J, Orava M, Tuomivaara L (2001). One versus two embryo transfer after IVF and ICSI: a randomized study. Hum Reprod.

[13] Dhont M (2001). Single-embryo transfer. Seminars in reproductive medicine.

[14] Gordts S, Campo R, Puttemans P, Brosens I, Valkenburg M, Norre J (2005). Belgian legislation and the effect of elective single embryo transfer on IVF outcome. Reprod Biomed Online.

[15] Kahn JA, von During V, Sunde A, Sordal T, Molne K (1993). The efficacy and efficiency of an in-vitro fertilization programme including embryo cryopreservation: a cohort study. Hum Reprod.

[16] Freemann L, Trounson A, Kirby C (1986). Cryopreservation of human embryos: progress on the clinical use of the technique in human in vitro fertilization. J In Vitro Fert Embryo Transf.

[17] McLernon DJ, Harrild K, Bergh C, Davies MJ, de Neubourg D, Dumoulin JC (2010). Clinical effectiveness of elective single versus double embryo transfer: meta-analysis of individual patient data from randomised trials. BMJ.

[18] Ercan CM, Kerimoglu OS, Sakinci M, Korkmaz C, Duru NK, Ergun A (2014). Pregnancy outcomes in a university hospital after legal requirement for single-embryo transfer. Eur J Obstet Gynecol Reprod Biol.

[19] Marek D, Langley M, Gardner DK, Confer N, Doody KM, Doody KJ (1999). Introduction of blastocyst culture and transfer for all patients in an in vitro fertilization program. Fertil Steril.

[20] Papanikolaou EG, Camus M, Kolibianakis EM, Van Landuyt L, Van Steirteghem A, Devroey P (2006). In vitro fertilization with single blastocyst-stage versus single cleavage-stage embryos. N Engl J Med.

[21] Gardner DK, Surrey E, Minjarez D, Leitz A, Stevens J, Schoolcraft WB (2004). Single blastocyst transfer: a prospective randomized trial. Fertil Steril.

[22] Styer AK, Wright DL, Wolkovich AM, Veiga C, Toth TL (2008). Single-blastocyst transfer decreases twin gestation without affecting pregnancy outcome. Fertil Steril.

[23] Heitmann RJ, Hill MJ, Richter KS, DeCherney AH, Widra EA (2013). The simplified SART embryo scoring system is highly correlated to implantation and live birth in single blastocyst transfers. J Assist Reprod Genet.

[24] Forman EJ, Upham KM, Cheng M, Zhao T, Hong KH, Treff NR (2013). Comprehensive chromosome screening alters traditional morphology-based embryo selection: a prospective study of 100 consecutive cycles of planned fresh euploid blastocyst transfer. Fertil Steril.

[25] Forman EJ, Hong KH, Franasiak JM, Scott RT (2014). Obstetrical and neonatal outcomes from the BEST Trial: single embryo transfer with aneuploidy screening improves outcomes after in vitro fertilization without compromising delivery rates. Am J Obstet Gynecol.

[26] Van Loendersloot L, Van Wely M, Limpens J, Bossuyt P, Repping S, Van Der Veen F (2010). Predictive factors in in vitro fertilization (IVF): a systematic review and meta-analysis. Hum Reprod Update.

[27] Practice Committee of American Society for Reproductive M, Practice Committee of Society for Assisted Reproductive T (2013). Criteria for number of embryos to transfer: a committee opinion. Fertil Steril.

[28] van Montfoort AP, Dumoulin JC, Land JA, Coonen E, Derhaag JG, Evers JL (2005). Elective single embryo transfer (eSET) policy in the first three IVF/ICSI treatment cycles. Hum Reprod.

[29] Veleva Z, Vilska S, Hyden-Granskog C, Tiitinen A, Tapanainen JS, Martikainen H (2006). Elective single embryo transfer in women aged 36–39 years. Hum Reprod.

[30] Niinimaki M, Suikkari AM, Makinen S, Soderstrom-Anttila V, Martikainen H (2013). Elective single-embryo transfer in women aged 40–44 years. Hum Reprod.

[31] Steinberg ML, Boulet S, Kissin D, Warner L, Jamieson DJ (2013). Elective single embryo transfer trends and predictors of a good perinatal outcome--United States, 1999 to 2010. Fertil Steril.

[32] van Montfoort AP, Fiddelers AA, Land JA, Dirksen CD, Severens JL, Geraedts JP (2007). eSET irrespective of the availability of a good-quality embryo in the first cycle only is not effective in reducing overall twin pregnancy rates. Hum Reprod.

[33] Group ECW (2000). Multiple gestation pregnancy. Hum Reprod.

[34] Grady R, Alavi N, Vale R, Khandwala M, McDonald SD (2012). Elective single embryo transfer and perinatal outcomes: a systematic review and meta-analysis. Fertil Steril.

[35] Min JK, Breheny SA, MacLachlan V, Healy DL (2004). What is the most relevant standard of success in assisted reproduction? The singleton, term gestation, live birth rate per cycle initiated: the BESST endpoint for assisted reproduction. Hum Reprod.

[36] Leiblum S, Kemmann E, Taska L (1990). Attitudes toward multiple births and pregnancy concerns in infertile and non-infertile women. J Psychosom Obstet Gynecol.

[37] Twisk M, van der Veen F, Repping S, Heineman M-J, Korevaar JC, Bossuyt PM (2007). Preferences of subfertile women regarding elective single embryo transfer: additional in vitro fertilization cycles are acceptable, lower pregnancy rates are not. Fertil Steril.

[38] Scotland G, McNamee P, Peddie V, Bhattacharya S (2007). Safety versus success in elective single embryo transfer: women’s preferences for outcomes of in vitro fertilisation. BJOG.

[39] Ryan GL, Sparks AE, Sipe CS, Syrop CH, Dokras A, Van Voorhis BJ (2007). A mandatory single blastocyst transfer policy with educational campaign in a United States IVF program reduces multiple gestation rates without sacrificing pregnancy rates. Fertil Steril.

[40] Medicine ASfR (1998). Practice Committee Opinion. Guidelines on number of embryos transferred.

[41] Stern JE, Cedars MI, Jain T, Klein NA, Beaird CM, Grainger DA (2007). Assisted reproductive technology practice patterns and the impact of embryo transfer guidelines in the United States. Fertil Steril.

[42] Maheshwari A, Griffiths S, Bhattacharya S (2011). Global variations in the uptake of single embryo transfer. Hum Reprod Update.

[43] Stillman RJ, Richter KS, Banks NK, Graham JR (2009). Elective single embryo transfer: a 6-year progressive implementation of 784 single blastocyst transfers and the influence of payment method on patient choice. Fertil Steril.

[44] Fiddelers AA, van Montfoort AP, Dirksen CD, Dumoulin JC, Land JA, Dunselman GA (2006). Single versus double embryo transfer: cost-effectiveness analysis alongside a randomized clinical trial. Hum Reprod.

[45] Veleva Z, Karinen P, Tomás C, Tapanainen JS, Martikainen H (2009). Elective single embryo transfer with cryopreservation improves the outcome and diminishes the costs of IVF/ICSI. Hum Reprod.

[46] Jain T, Harlow BL, Hornstein MD (2002). Insurance coverage and outcomes of in vitro fertilization. N Engl J Med.

[47] Boulet SL, Crawford S, Zhang Y, Sunderam S, Cohen B, Bernson D (2015). Embryo transfer practices and perinatal outcomes by insurance mandate status. Fertil Steril.

[48] Velez MP, Connolly MP, Kadoch IJ, Phillips S, Bissonnette F (2014). Universal coverage of IVF pays off. Hum Reprod.

